# Chronic exposure to a low dose of ingested petroleum disrupts corticosterone receptor signalling in a tissue-specific manner in the house sparrow (*Passer domesticus*)

**DOI:** 10.1093/conphys/cou058

**Published:** 2014-12-03

**Authors:** Christine R. Lattin, L. Michael Romero

**Affiliations:** Department of Biology, Tufts University, Medford, MA 02155, USA

**Keywords:** bird, endocrine disruption, glucocorticoid receptor, hypothalamic–pituitary–adrenal axis, mineralocorticoid receptor, toxicology

## Abstract

Stress-induced concentrations of glucocorticoid hormones (including corticosterone, CORT) can be suppressed by chronic exposure to a low dose of ingested petroleum. However, endocrine-disrupting chemicals could interfere with CORT signalling beyond the disruption of hormone titres, including effects on receptors in different target tissues. In this study, we examined the effects of 6 weeks of exposure to a petroleum-laced diet (1% oil weight:food weight) on tissue mass and intracellular CORT receptors in liver, fat, muscle and kidney (metabolic tissues), spleen (an immune tissue) and testes (a reproductive tissue). In the laboratory, male house sparrows were fed either a 1% weathered crude oil (*n* = 12) or a control diet (*n* = 12); glucocorticoid receptors and mineralocorticoid receptors were quantified using radioligand binding assays. In oil-exposed birds, glucocorticoid receptors were lower in one metabolic tissue (liver), higher in another metabolic tissue (fat) and unchanged in four other tissues (kidney, muscle, spleen and testes) compared with control birds. We saw no differences in mineralocorticoid receptors between groups. We also saw a trend towards reduced mass of the testes in oil-exposed birds compared with controls, but no differences in fat, kidney, liver, muscle or spleen mass between the two groups. This is the first study to examine the effects of petroleum on CORT receptor density in more than one or two target tissues. Given that a chronic low dose of ingested petroleum can affect stress-induced CORT titres as well as receptor density, this demonstrates that oil can act at multiple levels to disrupt an animal’s response to environmental stressors. This also highlights the potential usefulness of the stress response as a bioindicator of chronic crude oil exposure.

## Abstract (precis)

Studies of endocrine disruption rarely examine changes in hormone receptors. Chronic exposure to a low dose of ingested petroleum caused an increase in corticosterone receptors in subcutaneous fat, a decrease in liver, and no change in kidney, muscle, spleen, or testis, suggesting significant changes in corticosterone signaling.

## Introduction

Exposure to environmental toxicants can disrupt endocrine systems, including the vertebrate hypothalamic–pituitary–adrenal (HPA) axis responsible for the secretion of glucocorticoid hormones. At normal baseline concentrations, glucocorticoids are involved in essential processes, such as feeding behaviour and energy regulation (Landys *et al.*, 2006); at the increased concentrations caused by exposure to environmental perturbations, glucocorticoids play a key role in the stress response (Sapolsky *et al.*, 2000). In humans, disrupted glucocorticoid secretion causes health problems, including weight loss and fatigue (Dunlop, 1963), and animal studies have shown that individuals unable to mount a glucocorticoid response to stressors can die (Holmes *et al.*, 1979; Darlington *et al.*, 1990; Norris, 2000).

Even though glucocorticoids are very important in helping individuals to cope with environmental challenges, endocrine disruption of the HPA axis is generally understudied (Hinson and Raven, 2006). This is despite the fact that changes in glucocorticoid titres may be a useful bioindicator of chronic exposure to a variety of toxicants, from heavy metals (Norris, 2000; Franceschini *et al.*, 2009; Wada *et al.*, 2009) to polychlorinated biphenyls (Love *et al.*, 2003; Franceschini *et al.*, 2008; Iwanowicz *et al.*, 2009) to the focus of this study, crude oil (Rattner and Eastin, 1981; Gorsline and Holmes, 1982). Petroleum can be released into the environment via spills from tankers or pipelines, and it may persist for decades in wetland sediments (Burns *et al.*, 1994) and as surface and subsurface oil (Reddy *et al.*, 2002; Short *et al.*, 2004). Birds can ingest oil while preening oiled feathers or feeding, although the doses encountered by individuals in the wild are not well known (Leighton, 1993).

To assess the effects of ingested crude oil on the HPA axis in a controlled manner, we recently conducted a laboratory study of wild-caught house sparrows (*Passer domesticus*). House sparrows are excellent subjects for these kinds of toxicological studies for several reasons. First, they are easy to catch and do well in captivity, unlike many avian taxa, such as shorebirds (Serventy *et al.*, 1962). Second, because they are an invasive species in North America that competes directly with native bird species for nest sites and other resources, there is no negative impact, and potentially, even a mild beneficial impact, of removing them from the wild (Gowaty, 1984; Lowther and Cink, 2006). Third, as a passerine species, they are taxonomically similar to many birds living in coastal and riparian areas contaminated by oil, such as seaside sparrows (*Ammodramus maritimus*) and tree swallows (*Tachycineta bicolor*). Finally, the extensive validation data necessary for receptor binding studies are missing for most avian species, but are available for house sparrows (Breuner and Orchinik, 2009; Lattin *et al.*, 2012). In an earlier study, we found that 4 weeks of exposure to a 1% oil diet interfered with sparrows’ ability to elevate the glucocorticoid hormone corticosterone (hereafter CORT) in response to both a standardized stressor and an injection of adrenocorticotrophic hormone (Lattin *et al.*, 2014). This suggests that chronically oil-exposed animals may be characterized by a dampened response to acute stressors, perhaps due to adrenal dysfunction.

There are several ways in which endocrine-disrupting chemicals could interfere with CORT signalling in addition to disrupting hormone synthesis and secretion; these include affecting target cell uptake, receptor activation and binding to the promoters of target genes (Odermatt *et al.*, 2006). A complex mixture such as oil could potentially disrupt the HPA axis at multiple levels, and knowing more about the effects of petroleum on other aspects of CORT signalling may allow us to understand better why and how oil affects the stress response. Concentrations of CORT receptors are correlated with the magnitude of the downstream response on gene expression (Vanderbilt *et al.*, 1987; Yang *et al.*, 1989); therefore, if ingested oil decreases CORT receptor concentrations in metabolic tissues, such as muscle and fat, this would be consistent with the negative impact of oil being partly due to an inability to mobilize sufficient energy from these tissues to cope with stressors. However, few studies have looked beyond plasma hormone titres to the effects of toxicants on other mediators of hormone action.

In this study, we examined the effects of a chronic low dose of ingested petroleum on CORT receptors. We chose to examine receptors for two reasons. First, receptor binding is essential for creating a hormonal response (Beato and Sánchez-Pacheco, 1996). Second, in fish, CORT receptors have already been shown to be useful bioindicators of exposure to some toxicants. For example, rainbow trout (*Oncorhynchus mykiss*) exposed to high concentrations of waterborne copper showed decreased CORT receptor density in gill tissue (Dang *et al.*, 2000), and polychlorinated biphenyl-exposed Arctic char (*Salvelinus alpinus*) had decreased brain expression of CORT receptors (Aluru *et al.*, 2004). In birds, CORT functions primarily by binding to two intracellular receptors: the glucocorticoid receptor (GR), found ubiquitously throughout the body, and the mineralocorticoid receptor (MR), which has a slightly more limited distribution, found in high concentrations in the kidney, liver, brain, immune tissues and testis (Breuner and Orchinik, 2009; Schmidt *et al.*, 2010; Lattin *et al.*, 2012). Given that the MR has an approximately 10-fold higher affinity for CORT compared with the GR, it is thought that baseline CORT acts primarily via binding to the MR, whereas the actions of stress-induced CORT arise from binding to both the GR and the MR (de Kloet *et al.*, 1990, 1998).

We quantified GR and MR density in six different target tissues involved in energy balance and metabolism (liver, fat, muscle and kidney), the immune system (spleen) and reproduction (testes) in male house sparrows fed either a 1% oil (*n* = 12) or a control diet (*n* = 12) for 6 weeks. Studies of CORT receptors in fish have mostly shown either a decrease or no change in receptor density in toxicant-exposed animals compared with healthy animals (Dang *et al.*, 2000; Aluru *et al.*, 2004; Aluru and Vijayan, 2004; Gravel and Vijayan, 2006); therefore, we predicted that we would also see unchanged or lower receptor concentrations in sparrows consuming an oiled diet. Unchanged receptor concentrations combined with the lower stress-induced CORT titres previously seen in sparrows exposed to a low dose of ingested petroleum (Lattin *et al.*, 2014) would indicate an overall reduction in the stress response compared with control sparrows. Decreased receptor density combined with decreased stress-induced CORT titres would potentially amplify this reduction.

## Materials and methods

### Study animals and experimental diets

Wild house sparrows (*n* = 24) were caught between 28 January and 5 Feburary 2013 in Medford, MA, USA using seed-baited Potter traps and mist nets at bird feeders. As we wished to examine receptors in gonadal tissue without decreasing the sample size, we only used male birds in this study. It is important to note that because male birds cannot depurate lipophilic contaminants into eggs (Ewins *et al.*, 1999), the effects in males may be larger than what we would expect to see in females. Birds were initially housed together in an outdoor aviary and were transferred to bird rooms indoors on 6 Feburary 2013, where they were housed singly in natural day-length conditions. Both outdoors and indoors, sparrows had *ad libitum* access to water, grit and mixed seeds. After 2 weeks to adjust to laboratory conditions, sparrows were switched to experimental diets.

For the oil diet, we used a dose of 1% oil weight:food weight based on a pilot study demonstrating that 5 weeks of exposure to this dose, but not to 0.1 or 0.01% doses, significantly reduced stress-induced CORT in house sparrows (Lattin *et al.*, 2014). Gulf of Mexico Sweet Louisiana crude oil was weathered to ∼75% of its original volume by heating at a low temperature and stirring continuously. This weathering treatment is likely to have dispersed the most toxic volatiles, which do not persist for very long in the environment (Chen and Denison, 2011). Weathered crude was combined with an equal volume of organic sunflower oil (Catania-Spagnia Corporation, Ayer, MA, USA) to facilitate mixing into de-husked millet (Agway, Grandin, ND, USA) for a total volume of 2 ml petroleum and sunflower oil/100 g food. The control diet consisted of sunflower oil mixed into de-husked millet instead of the petroleum. We randomly chose half of the birds (*n* = 12) to receive the oil diet and the other half (*n* = 12) to receive a control diet. Birds fed freely, without gavage or other force-feeding techniques, which could potentially have their own effects on CORT signalling. Although we did not measure food consumption, there were no body mass differences between birds on the oil and control diets after 2 or 4 weeks (Lattin *et al.*, 2014), so all birds fed to maintain body weight. To compensate for their low-diversity diets, sparrows also received Nekton-S multi-vitamin supplement for cage birds (Günter Enderle, Pforzheim, Germany) at manufacturer-recommended concentrations (0.4/100 g of diet).

As part of another study published previously (Lattin *et al.*, 2014), we took body mass measurements and blood samples from all birds immediately before the onset of feeding and 2 and 4 weeks into the feeding experiment. The results of this sampling have been described in detail elsewhere (Lattin *et al.*, 2014). All procedures were performed according to Association for Assessment and Accreditation of Laboratory Animal Care (AAALAC) guidelines, and all protocols were approved by the Tufts University Animal Care and Use Committee (protocol #M2012-160).

### Chemical adrenalectomy with mitotane

Radioligand binding assays for quantifying receptors require the absence of circulating CORT. Rather than having the adrenal glands removed surgically, the birds were chemically adrenalectomized with two injections of mitotane (ortho, para-DDD; Breuner *et al.*, 2000). Mitotane appears to inhibit CORT production by suppressing mitochondrial steroid 11β-hydroxylase and cholesterol side-chain cleavage activity selectively in the zona fasciculata of the adrenals (Sanderson, 2006). In house sparrows, mitotane has been shown to be both reversible (stress-induced CORT levels recovered by 10 days after a mitotane injection) and specific in its actions (mitotane treatment did not affect testicular weights or testosterone in house sparrows; Breuner *et al.*, 2000).

Approximately 36 and 24 h before the birds were killed, mitotane (180 mg/kg body weight) was dissolved in peanut oil and injected into the pectoralis muscle of both oil-exposed and control animals (Breuner *et al.*, 2000; Lattin *et al.*, 2012). To measure the success of the mitotane treatment, on the morning of sacrifice (∼36 h after the first mitotane injection), animals were restrained in cloth bags for 30 min and blood samples of ∼30 µl taken from the brachial vein using heparinized microcapillary tubes. Whole blood samples were kept on ice until centrifuged 2–4 h later; we then drew off and froze the plasma until radioimmunoassay.

Radioimmunoassays were done following Wingfield *et al.* (1992), using antibody B3-163 (Esoterix, Calabasas Hills, CA, USA). All samples were run in the same assay. Average recovery was 84%, detectability was 1 ng CORT/ml plasma, and the intra-assay coefficient of variation was 3%. Mitotane successfully reduced stress-induced CORT for both oil-exposed birds (Student’s paired *t*-test: *t* = −7.1, d.f. = 11, *P* < 0.0001) and control birds (Student’s paired *t*-test: *t* = −10.5, d.f. = 11, *P* < 0.0001). Mean CORT (±SD) after mitotane treatment was 1.4 ± 3.4 ng/ml, in comparison to previous values of 17.2 ± 8.0 ng/ml for oil-exposed birds and 32.8 ± 12.8 ng/ml for control birds (Lattin and Romero, 2014).

### Receptor binding assays

We killed the sparrows after 6 weeks on experimental diets in order to ensure that the birds had a chance to recover from the effects of blood sampling at 4 weeks (it is recommended that researchers remove no more than 1% of an animal’s body weight in blood every 2 weeks; Harr, 2002). The birds were deeply anaesthetized using intramuscular injections of ketamine (∼80 mg/kg body weight) and xylazine (∼20 mg/kg body weight), at doses appropriate for this species (Muresan *et al.*, 2008). We then perfused the animals transcardially with ice-cold heparinized saline, and extracted and flash-froze whole liver, left pectoralis, spleen, subcutaneous fat from the furcula, kidneys and testes using dry ice. We always collected tissues in the same order; the time to extract all tissues was ≤14.5 min after death (mean time ± SD, 12.2 ± 1.1 min). Tissues were stored at −80°C until assayed.

We used radioligand binding assays to quantify CORT receptor concentrations in tissue following Breuner and Orchinik (2001) and Lattin *et al.* (2012). Briefly, on the day of assay, tissues were thawed, homogenized and spun in an ultracentrifuge. The infranatant (for fat) or supernatant (for other tissues) was incubated with 10 nm [^3^H]CORT and one of the following: (i) buffer, to measure total binding; (ii) excess unlabelled CORT, to measure non-specific binding; or (iii) excess RU486 (mifepristone), which binds only the GR. After subtracting non-specific binding, MR binding can be calculated directly from test tubes containing RU486; GR binding can be calculated by subtracting MR binding from total binding. Based on affinity estimates from previously published saturation experiments (Lattin *et al.*, 2012), mass action predicts that 10 nm [^3^H]CORT should occupy >95% of MRs and ∼63% of GRs. Each point sample was run in triplicate. Samples were incubated at optimized temperatures and times for each tissue (Lattin *et al.*, 2012).

Incubations were terminated by filtration over Whatman GF/B filters in a Brandel harvester, and rinsed three times with 3 ml of ice-cold buffer. Filter paper was mixed with scintillation fluid and assayed on a scintillation counter. Binding in individual samples was standardized per milligram of protein using Bradford assays with bovine serum albumin standards.

### Chemicals

Gulf of Mexico Sweet Louisiana crude was obtained from British Petroleum Exploration and Production Inc. (Houston, TX, USA). Mitotane, sodium heparin, corticosterone, bovine serum albumin and Bradford reagent were purchased from Sigma Aldrich (St Louis, MO, USA), RU486 from Tocris Bioscience (Minneapolis, MN, USA) and Ultima Gold scintillation fluid and [^3^H]CORT from Perkin Elmer (Waltham, MA, USA). Xylazine was from Akorn, Inc. (Decatur, IL, USA) and ketamine from Fort Dodge Animal Health (Fort Dodge, IA, USA).

### Data analysis

Samples containing 1–10 mg/ml protein give accurate results in corticosteroid receptor radioligand binding assays (López Bernal *et al.*, 1984). However, four samples fell below this 1 mg/ml cut-off value and were excluded from analysis (spleen, one control and one experimental male; testes, two experimental males). As a result of differences in homogenization technique and incubation time and temperature among different tissues, receptor binding values for different tissues cannot be compared directly, so we ran each tissue in a separate analysis. For receptor analyses, we compared GR or MR concentrations for each tissue between birds on a 1% oil diet and control birds using analysis of variance (ANOVA). These analyses are fairly robust to violations of normality assumptions, but not to violations of homogeneity of variance among groups (Day and Quinn, 1989); therefore, for each analysis, we used Levene’s test to ensure homogeneity of variances (Quinn and Keough, 2002) and used a Welch’s ANOVA in situations where variances among groups were not homogeneous (Day and Quinn, 1989).

We used two-sided Student’s *t*-tests to compare tissue mass between sparrows on a 1% diet and control animals, after standardizing all tissue mass measures by total body mass to control for part–whole correlations (Christians, 1999). Previous studies in ducks (*Anas platyrhynchos*) have shown that ingested petroleum negatively affects male fertility (Holmes and Cavanaugh, 1990) and can cause increased liver mass and decreased spleen mass (Lee *et al.*, 2012); Because of this, we specifically predicted that 6 weeks of exposure to a 1% oil diet would cause testes and spleen to shrink and liver to grow. For fat, muscle and kidney, we had no directional predictions. All statistical analyses were run using JMP 10.0 (SAS Institute, Cary, NC, USA).

## Results

**Figure 1:**
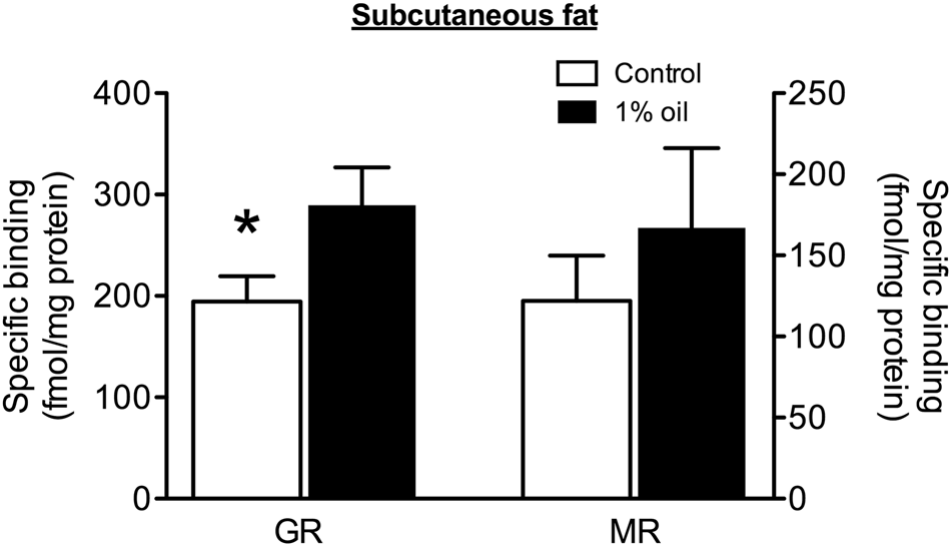
Point sample analysis of glucocorticoid receptors (GRs; left *y*-axis) and mineralocorticoid receptors (MRs; right *y*-axis) in subcutaneous fat from the furcula in male house sparrows on a diet containing 1% weathered crude oil (*n* = 12; filled bars) or a control diet (*n* = 12; open bars). Data represent means ± SEM of specific binding of 10 nm [^3^H]corticosterone to house sparrow cytosol, standardized by protein concentration. Significant differences between birds on oil and control diets are indicated (*P < 0.05).

In subcutaneous fat, GR receptor density was significantly higher in animals exposed to a 1% oil diet compared with control animals (Fig. 1; *F*_1,22_ = 4.46, *P* = 0.046; Levene’s test, *P* = 0.19); however, there were no differences in MR density between the two groups (Fig. 1; *F*_1,22_ = 0.63, *P* = 0.43; Levene’s test, *P* = 0.077). Although inspection of Fig. 1 suggests that MRs also increased with oil consumption, the mean was influenced by one individual in the oil group with much higher MR binding than the other individuals in that group; without that individual, the means are nearly identical (122 vs. 131 fmol/mg protein).

**Figure 2:**
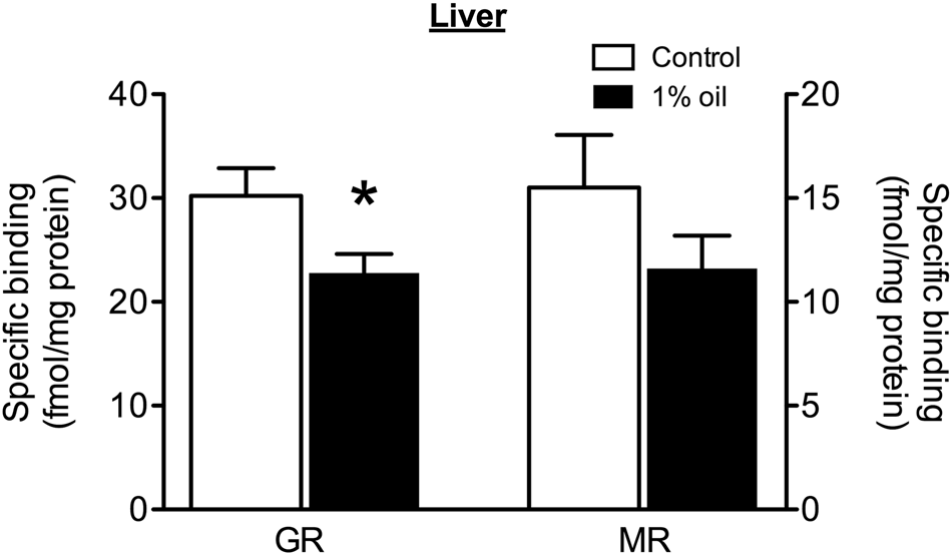
Point sample analysis of glucocorticoid receptors (GRs; left *y*-axis) and mineralocorticoid receptors (MRs; right *y*-axis) in whole liver in male house sparrows on a diet containing 1% weathered crude oil (*n* = 12; filled bars) or a control diet (*n* = 12; open bars). Data represent means ± SEM of specific binding of 10 nm [^3^H]corticosterone to house sparrow cytosol, standardized by protein concentration. Significant differences between birds on oil and control diets are indicated (*P < 0.05).

In liver, GR density was significantly lower in animals exposed to a 1% oil diet compared with control animals (Fig. 2; *F*_1,22_ = 5.16, *P* = 0.033; Levene’s test, *P* = 0.20), but again, MR density did not differ between the two groups (Fig. 2; *F*_1,18.5_ = 1.72, *P* = 0.21; Levene’s test, *P* = 0.043).

**Figure 3:**
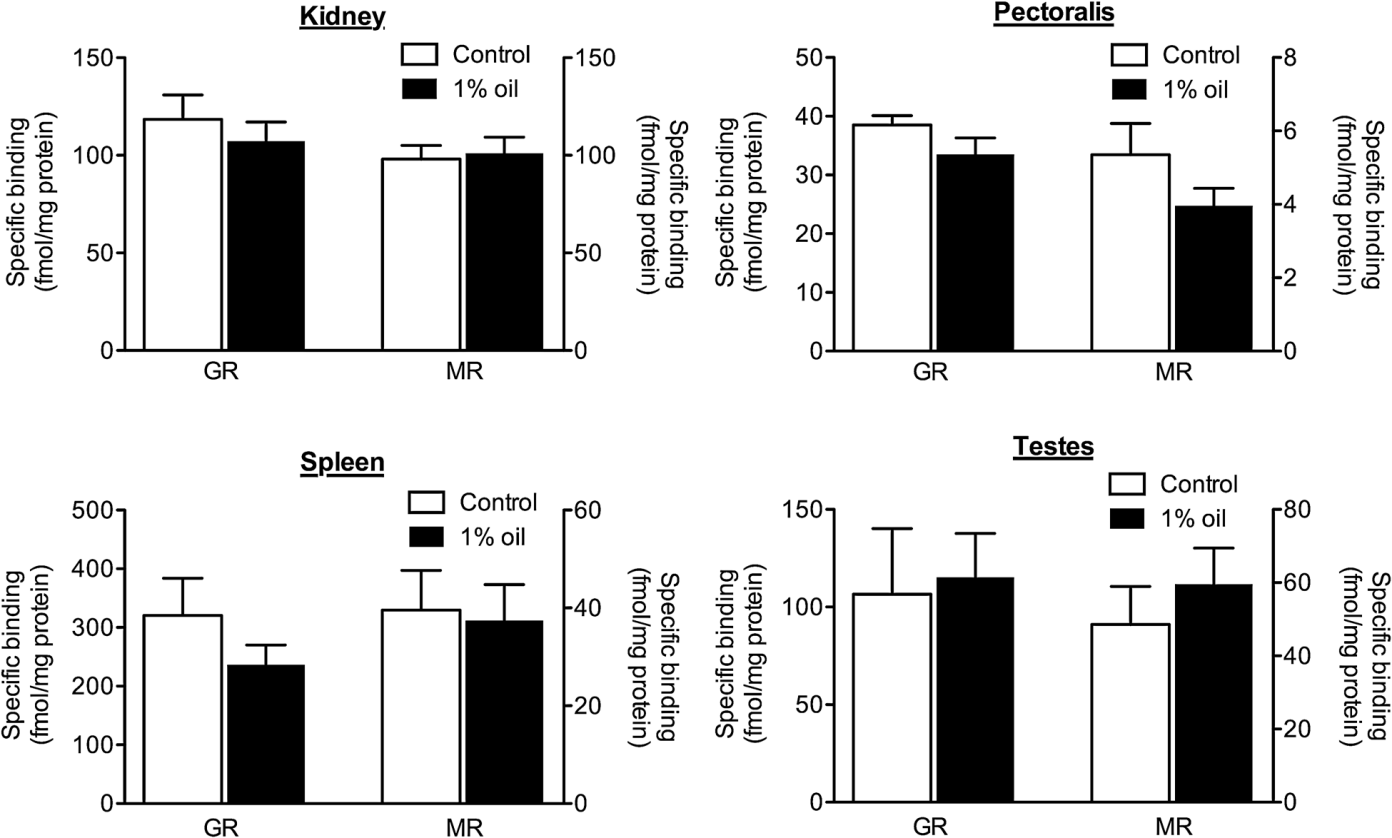
Point sample analysis of glucocorticoid receptors (GRs; left *y*-axes) and mineralocorticoid receptors (MRs; right *y*-axes) in kidneys (top left), right pectoralis muscle (top right), spleen (bottom left) and testes (bottom right) in male house sparrows on a diet containing 1% weathered crude oil (*n* = 12; filled bars) or a control diet (*n* = 12; open bars). Data represent means ± SEM of specific binding of 10 nm [3H]corticosterone to house sparrow cytosol, standardized by protein concentration.

There were no differences in CORT receptor concentrations between animals on a 1% oil diet and control animals in kidney (Fig. 3; GR: *F*_1,22_ = 0.49, *P* = 0.49; Levene’s test, *P* = 0.33; and MR: *F*_1,22_ = 0.087, *P* = 0.77; Levene’s test, *P* = 0.45), pectoralis muscle (Fig. 3; GR: *F*_1,22_ = 2.40, *P* = 0.14; Levene’s test, *P* = 0.064; and MR: *F*_1,22_ = 2.03, *P* = 0.17; Levene’s test, *P* = 0.29), spleen (Fig. 3; GR: *F*_1,20_ = 1.39, *P* = 0.25; Levene’s test, *P* = 0.074; and MR: *F*_1,20_ = 0.039, *P* = 0.85; Levene’s test, *P* = 0.97) or testes (Fig. 3; GR: *F*_1,20_ = 0.042, *P* = 0.84; Levene’s test, *P* = 0.25; and MR: *F*_1,20_ = 0.57, *P* = 0.46; Levene’s test, *P* = 0.85).

Tissue mass did not differ between oil-exposed and control animals for any tissue (Table 1). However, animals on a 1% oil diet tended to have smaller testes mass than control animals (Table 1).

**Table 1:** Tissue masses of six different tissue types from male house sparrows (*Passer domesticus*) fed a control diet (*n* = 12) or a diet mixed with 1% weathered crude oil (*n* = 12)

Tissue type	Control mean mass (g)	Oil-exposed mean mass (g)	*t*-Ratio	*P*-Value
Fat	0.089 ± 0.012	0.11 ± 0.014	0.46	0.65
Kidney	0.19 ± 0.0052	0.20 ± 0.0077	0.95	0.35
Liver	0.80 ± 0.024	0.87 ± 0.029	1.61	0.12
Muscle	1.66 ± 0.044	1.74 ± 0.062	0.87	0.39
Spleen	0.028 ± 0.0039	0.029 ± 0.0041	0.17	0.86
Testes	0.18 ± 0.048	0.071 ± 0.034	−1.97	0.062

The raw means ± SEM for each group are shown; statistics were run on tissue means corrected for total body mass.

## Discussion

In house sparrows exposed to a 1% dose of weathered crude oil for 6 weeks, the GR concentration was lower in one metabolic tissue (liver), higher in a second metabolic tissue (subcutaneous fat) and not different in four other tissues (kidney, muscle, spleen and testes) compared with control animals. We saw no significant differences in MRs between the two groups, although the trends for MRs went in the same direction as the GR differences for both liver and subcutaneous fat. The overall differences between effects on GRs and MRs may be consistent with chronic low doses of ingested petroleum having greater negative impacts on the acute CORT response (thought to be largely mediated by the GR), rather than on baseline effects (thought to be mediated by the MR; Romero, 2004; Landys *et al.*, 2006).

With the exception of fat tissue, these results support our general hypothesis that sparrows ingesting the oil diet would have reduced or unchanged CORT receptor density in target tissues, as in previous studies in fish (Dang *et al.*, 2000; Aluru and Vijayan, 2004; Aluru *et al.*, 2004; Gravel and Vijayan, 2006). As the same group of animals also had lower acute CORT titres in response to both a standardized stressor and an adrenocorticotrophic hormone challenge after 4 weeks on a 1% oil diet (Lattin *et al.*, 2014), this suggests that the overall tissue response to stressors may have been generally reduced (kidney, muscle, spleen and testes) or greatly reduced (liver) in impacted animals compared with control animals. Given that the CORT receptors in different tissues responded heterogeneously to a low dose of ingested oil, our data suggest that it may be necessary to examine receptor density in multiple tissue types in order to understand the effects of petroleum on the stress response of wild animals. It is important to note, however, that there are a number of other physiological mediators, including metabolizing enzymes (Chapman *et al.*, 2013), plasma binding globulins (Breuner *et al.*, 2012) and local hormone production (Schmidt *et al.*, 2008), that can affect a the response of a given tissue to CORT, and we did not quantify those mediators here or assess changes in the downstream response. It should also be noted that house sparrows, a small terrestrial passerine and human commensal, may not be representative of all avian species chronically exposed to oil, such as shorebirds, ducks and gulls. However, the fact that ingested crude oil suppressed stress-induced CORT titres in both mallard ducks (Gorsline and Holmes, 1982) and house sparrows (Lattin *et al.*, 2014) suggests that the effects of petroleum on the physiological stress response may be conserved across avian taxa.

Hormone receptors are typically regulated at least partly by circulating hormone titres (Sapolsky *et al.*, 1984; Reul *et al.*, 1987; Spencer *et al.*, 1991; Kalman and Spencer, 2002). For example, rats had increased brain and spleen GR 6 days after adrenalectomy, a rise which could be blocked with replacement doses of hormone (Spencer *et al.*, 1991). However, our results indicate that CORT receptors in sparrows were generally not up-regulated in response to the suppression of the acute CORT response caused by ingested oil (Lattin *et al.*, 2014). This could have major negative consequences on the ability of the animals to survive stressors, such as exposure to an extreme weather event or a predator attack; an overall general suppression of the stress response may be part of the reason why petroleum-exposed animals show increased mortality after exposure to subsequent stressors (Holmes *et al.*, 1979).

These receptor data also suggest that oil-exposed animals may alter how they mobilize energy in response to stressors. It is possible that decreased liver GR density and increased fat GR density in oil-exposed animals could lead to decreased mobilization of glucose and increased mobilization of fatty acids in stressed animals. In a previous experiment, we found no differences in baseline plasma glucose between control and oil-exposed birds after 4 weeks of oil feeding (Lattin *et al.*, 2014); we also collected stress-induced blood samples in that study, but unfortunately all plasma was used in hormone assays. Thus, it remains to be seen whether there might be differences in plasma glucose or other metabolites in impacted animals after stressor exposure.

The exact mechanism for oil-induced disruption of CORT receptor density remains to be clarified. Different toxicants have different types of effects on CORT receptors; for example, arsenic can alter the ability of GRs to regulate gene transcription (Bodwell *et al.*, 2004), whereas polychlorinated biphenyls appear to compete with CORT for binding to the GR and act as GR antagonists (Johansson *et al.*, 1998, 2005). Sometimes, the direction of these effects depends upon the toxicant dose and the cellular level of activated CORT receptors (Bodwell *et al.*, 2004). It seems likely that some of the GR effects we saw may be due to activation of the aryl hydrocarbon receptor, which is bound by petroleum components (Denison and Nagy, 2003), is known to have cross-talk with the GR (Dvorák *et al.*, 2008) and can modulate the function of the GR as a transcription factor (Ohtake *et al.*, 2009). In rainbow trout, stimulation of the aryl hydrocarbon receptor inhibited liver GR responsiveness to circulating CORT (Aluru and Vijayan, 2004).

We also compared overall tissue mass in oil-exposed and control animals for each of these six tissues. Male sparrows exposed to a 1% oil diet for 6 weeks tended to have smaller testes than control males, although this was not significant. The finding of smaller testes with oil exposure is consistent with previous studies demonstrating negative effects of petroleum on avian reproduction (Holmes and Cavanaugh, 1990; Leighton, 1993). We saw no significant differences in the mass of any other tissues between oil-exposed and control males. These results differ from a previous study that found larger livers and smaller spleens in mallard ducks dosed with fuel oil for 5 days (Lee *et al.*, 2012). However, not all studies using oil feeding protocols in birds find greater liver mass in exposed animals compared with control animals (Pattee and Franson, 1982; Peakall and Hallett, 1982). These differences among studies may be due to experimental differences in oil composition, dose, time course or species used. Also, simply because we did not see significant differences in liver and spleen mass between the two groups, it does not mean that organ function was not negatively impacted by exposure to ingested crude oil. For example, mallard ducks orally dosed with oil for 4 weeks were less able to resist infection by a bacterial pathogen compared with control ducks, even though there were no differences in the number of antibody-secreting cells in the spleen between the two groups (Rocke *et al.*, 1984).

This is the first study to examine the effects of petroleum on CORT receptor density in more than one or two target tissues. To our knowledge, it is the first study of CORT receptor changes in response to chronic toxicant exposure in any bird species. We believe that makes this a particularly important contribution to our understanding of oil as an endocrine-disrupting chemical, especially given the heterogeneous response we saw in GR concentrations in different tissue types. Given that a chronic low dose of ingested petroleum not only affects stress-induced CORT titres, but also receptor density, this demonstrates that oil can act at multiple levels to disrupt an animal’s response to environmental stressors. Because we saw changes in both CORT secretion and CORT receptors in oil-exposed birds without seeing concomitant changes in body mass or a number of different blood chemistry parameters (Lattin *et al.*, 2014), this work also highlights the potential usefulness of the stress response as a bioindicator of chronic crude oil exposure.

## Funding

This work was supported by the National Science Foundation [IOS-1048529 to L.M.R.], the Tufts Institute for the Environment [to C.R.L.] and the Environmental Protection Agency's Science to Achieve Results (STAR) fellowship program [FP-91735001 to C.R.L.].
